# Alzheimer and depressive cognitive-like behaviors in male and female rats: A new method for exposure to ambient air pollution

**DOI:** 10.1016/j.mex.2019.03.018

**Published:** 2019-03-28

**Authors:** Saeed Motesaddi Zarandi, Abbas Shahsavani, Fariba Khodagholi, Yadolah Fakhri

**Affiliations:** a Department of Environmental Health Engineering, School of Public Health and Safety, Shahid Beheshti University of Medical Sciences, Tehran, Iran; b Environmental and Occupational Hazards Control Research Center, Shahid Beheshti University of Medical Sciences, Tehran, Iran; c Neuroscience Research Center, Shahid Beheshti University of Medical Sciences, Tehran, Iran; d Department of Environmental Health Engineering, Student Research Committee, School of Public Health and Safety, Shahid Beheshti University of Medical Sciences, Tehran, Iran

**Keywords:** In-vivo model, Alzheimer, Depressive and Cognitive-like behaviors, PM_2.5_, In-vivo model, Alzheimer, Depressive, Cognitive-like behaviors, Rats

## Abstract

In the previous studies regarding the effects of exposure to ambient air pollution on biological markers and/or behavior of animals, the gaseous pollutants are not separated from the particulate matter (PM). Hence the synergetic effect of gaseous pollutants and PM was not considered. In this regard, current study was aimed to devolve a new method for separation of PM from gaseous pollutants. Also, the effect of exposure to fine particulate matter (PM_2.5_) on the Alzheimer and depressive cognitive-like behaviors in rats after 3 and 6 months were investigated. Three chambers were designed including exposure group 1 (PM_2.5_ plus gaseous pollutants alone), exposure group 2 (gaseous pollutants alone) and control group (clean air). Exposure time was 5 h per day (9.00 a.m.–2.00 p.m.) for 4 days per week. The concentration of PM_2.5_ and gaseous pollutants (O_3_, NO_2_, and SO_2_) were monitored in the exposure hours, continuously. Concentration of PM_2.5_ by beta attenuation method and concentration of O_3_, NO_2_, and SO_2_ by UV fluorescence was monitored. Also, the concentration of metals including Al, Cr, Mn, Pb, Cd, Ni, Fe, and Cu and 16-polycyclic aromatic hydrocarbons (PAHs) bound PM_2.5_ by inductively coupled plasma mass spectrometry (ICP-MS) and gas chromatography-mass spectrometry (GC—MS) were analyzed, respectively. Cognitive-like behavior related to Alzheimer and depressive behaviors were determined by Y maze and Force swimming. The concentration of PM_2.5_ in the 3 and 6 months exposure was higher than WHO guideline, significantly (p-value <0.05). The concentration of O_3_, NO_2_ and SO_2_ in the 3 and 6 months exposure was lower than WHO guideline, significantly (p-value <0.05). The order of metals in the PM_2.5_ according to mean concentration Al > Ca > Cu > Cd > Na > Fe > Cr > Ni > Mn > Pb. Also, the sum concentration of 16-PAHs in the PM_2.5_ in the 3 and 6 months exposure was 45.7 ± 37.15 ng/m^3^ and 30.04 ± 25.27 ng/m^3^, respectively. Exposure to PM_2.5_ cannot significantly increase Alzheimer and depressive cognitive-like behaviors in the rats. Also, a significant difference between male and female in Alzheimer and depressive cognitive-like behaviors not observed.

• A new method for separation of PM_2.5_ from other PM in the ambient air by ECO-PM sampler was presented.
• A new method for separation of PM_2.5_ from gaseous pollutants in the ambient air by HEPA filter and active carbon was presented.
• Tow exposure groups including exposure 1: PM_2.5_ plus gaseous pollutants and exposure 2: gaseous pollutants only were designed for increased accuracy of the in-vivo study.
• Exposure to PM_2.5_ cannot cause significant increased Alzheimer and depressive cognitive-like behaviors in the rats.

## Graphical abstract

**Figure:**
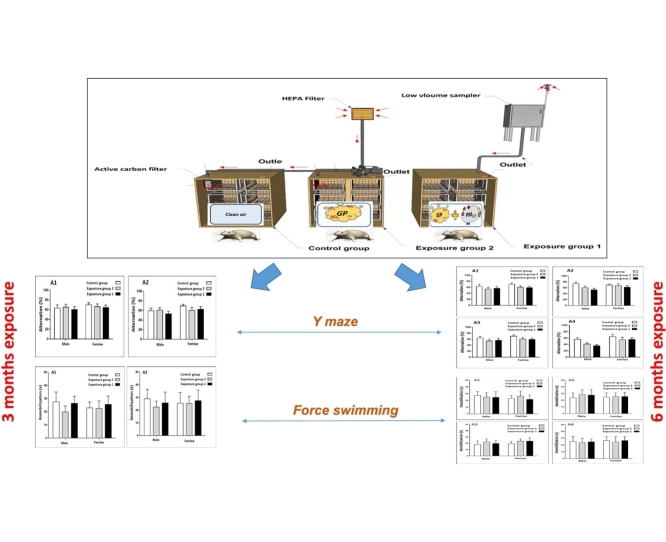

**Specifications Table**

**Table:** 

Subject area:	Environmental Science
More specific subject area:	A new method of exposure to Air pollution in the in-vivo model study.
Method name:	In-vivo model; Alzheimer; Depressive and Cognitive-like behaviors
Reagents/tools:	Y-maze and forced swimming
Experimental design:	Alzheimer and Depressive like behaviors by Y-maze and force swimming was investigated, and concentration of PM_2.5_, O_3_, SO_2_ and NO_2_, PAHs and metals bound to PM_2.5_ based on approved methods was detected during the exposure time.
Trial registration:	Not applicable
Ethics:	It was approved
*Value of the Protocol:	• The data presented in this article can be useful for managers of air pollution in the urban areas and health management organization. • Due to adverse health effect of air pollution and its consequence on human health effect and increases prevalence Alzheimer and depressive symptoms in the urban areas, investigation on the effect of exposure to air pollution on the Alzheimer and depressive cognitive-like behaviors is necessary.

## Method details

Air pollution is a complex mixture of gases, particulate matter (PM), metals and organic compounds [1]. Recently global concerns increased due to the adverse effects of air pollution on human health [2].

In this regard, the International Agency for Research on Cancer (IARC) declared that air pollution is definitive carcinogenic (Group 1) for humans [3]. The relationship between the exposure to air pollution and autoimmune diseases [4], cardiovascular diseases [5], asthma [6], chronic obstructive pulmonary disease (COPD) [7], and cognitive disorders [8] were well approved.

PM classified as the ultrafine (≤0.1 μm), fine (≤2.5 μm) and coarse (2.5–10 μm), and particles [9]. Due to disperse uniformly, remaining as suspended in the air for a long period time, capability of penetration into the deepest respiratory system exposure to PM_2.5_ is a matter of concern [10]. Also, due to the high surface area of PM_2.5,_ it can bind with metals, heavy metals, polycyclic aromatic hydrocarbons (PAHs), metalloids, elemental carbon, organic carbon, ammonium, sulfate, nitrate, viruses, bacteria, which could increase adverse health effects [11]. Approximately 7.6% (42 million) of the total number of deaths worldwide is related to Exposure to PM_2.5_ [12].

PAHs in the air and bound PM_2.5_ have mutagenic and/or carcinogenic properties [13] and the metals include chromium (Cr), lead (Pb), arsenic (As), cadmium (Cd), nickel (Ni), manganese (Mn) and copper (Cu) bound with PM_2.5_ induced to oxidative stress [14], following with an increase in DNA lesions [15].

The association between air pollution and neuroinflammatory and neurodegenerative as one of the routes for increasing neurodegenerative disorders (ND) was approved [16,17]. However, pathophysiological mechanisms on the central nervous system (CNS) due to PM exposure is elucidated [18]. Alzheimer’s disease (AD) is progressive and irreversible ND that is most observable in older adults peoples [19]. Almost 30 million persons in the worldwide and more than 4 million in the USA are suffered by the AD [20]. Advanced age is of the main pathogenic factors for AD. However, other common pathogenic factors are included such as family history [21], sex [22], down syndrome [23], cardiovascular disease [24], poor educational level and environmental pollutants [25]. Also, in some societies, Alzheimer’s like behaviors has been associated with air pollution exposure [26]. Exposure to air pollution can be related to depressive diseases [27]. Potential biochemical mechanisms relating to depressive diseases is including oxidative stress; dysfunction of neurotransmitter systems; alterations of neurohumoral, and autonomic regulation [28]. Previous empirical studies have shown that exposure to air pollution can be increases the prevalence of depressive diseases in the Netherlands, Korea and Japan [29]. Also, an increasing number of emergency visits due to depression in Korea and Canada have been observed [30].

In previous investigations regarding the effects of exposure to ambient air pollution on biological markers and/or behavior of animals, the gaseous pollutants are not separated from the particulate matter (PM). Hence, the synergetic effect of gaseous pollutants and PM was not considered. Previous research demonstrated that exposure to ambient PM following gaseous pollutants can have a synergetic effect on the health animals [31,32]. Therefore perform of study on effect of exposure to ambient PM and gaseous pollutants separately is necessary.

Hence, in the current study, a new method for separation of ambient PM from gaseous pollutants was presented. Also, changes of Alzheimer and depressive-like behaviors in the male and female rats due to inhalation PM_2.5_ in ambient air by Y-maze and Force swimming cognitive-like behavior tests were investigated.

## Material and methods

### Rats

Ninety-six six-week-old male (n = 48) and female (n = 48) Wistar rats (with an average weight of 85 ± 10 g) were purchased from Pasteur Institute, Tehran, Iran. Rats were maintained in the laboratory standard conditions including supplying the water, food, libitum, and light (12 h)/dark (12 h) cycles. Also, before the starting study, rats were maintained for one week in similar environmental conditions including temperature (20–25 °C) and relative humidity (40–60%). The protocols of the current study were confirmed by the laboratory animal research principles by the Animal Welfare Act [33]. Ethics approval by Shahid Beheshti University of Medical Sciences Ethics Committee and the number of rats were minimized as far as possible.

### Study location and method of exposure

The pilot animal room is located in the roof school of public health in Shahid Beheshti University (35.7991°N, 51.3947°E) at an altitude of ˜20 m above the ground.

**Fig. 1:**
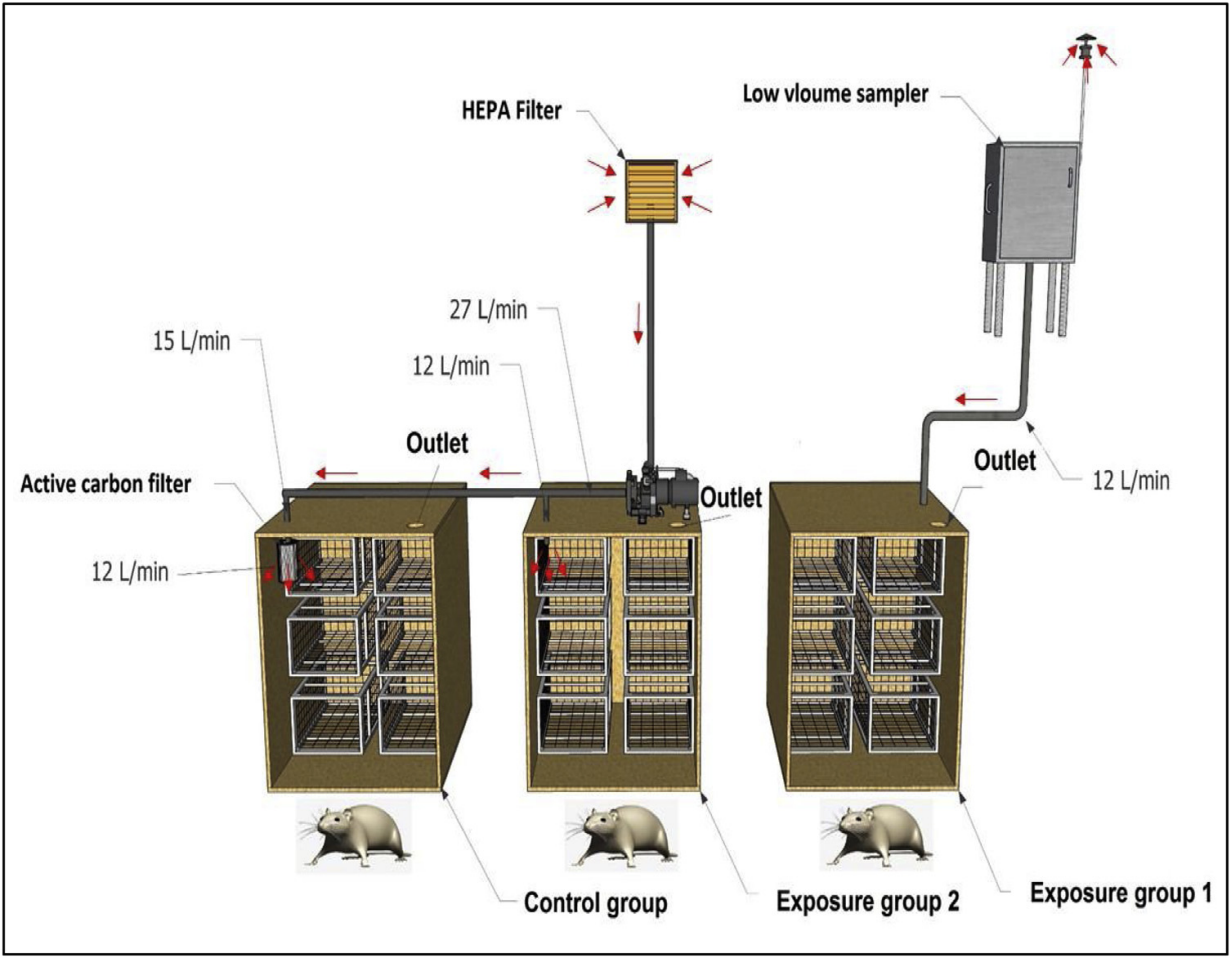
Schematic of exposure method. Three chambers include exposure group 1 (PM_2.5_ plus GP), exposure group 1 (GP alone) and control group (clean air).

In the pilot animal room three chambers were designed including exposure group 1 (PM_2.5_ plus gaseous pollutants alone), exposure group 2 (gaseous pollutants alone) and control group (clean air). Exposure group 1: Urban ambient air with 12 l/min flow rate was introduced into the chamber by Echo PM (TCR Tecora Italy) low volume sampler (LVS) without sampling filter. It is noteworthy that LVS was designed for sampling of PM in the ambient air (PM_10_ and PM_2.5_), but when the sampling filter removed, outlet air will contain PM (Fig. 1) [34]. In the current study, PM_2.5_ warhead was used; hence, output air contains ambient PM_2.5_ plus gaseous pollutants. Exposure group 2: urban ambient air with 12 l/min flow rate was passed into the chamber by a vacuum pump (Model LFS-113; Gillian Instrument Corp.). For removing of PM, HEPA filter model H13 is located in the inlet valve of the vacuum pump. HEPA filter class H13 (Camfill-FARR, Switzerland) removes PM with 99.97%. Therefore, outlet air contains gaseous pollutants alone. Control group: In order removing PM and gaseous pollutants, ambient air with 12 l/min flow rate was subjected into the chamber by a vacuum pump with HEPA filter model H13 (SungJin Co., Ltd., Korea) and active carbon air filter in the inlet and outlet valves, respectively (Fig. 1).

### Exposure periods

**Fig. 2:**
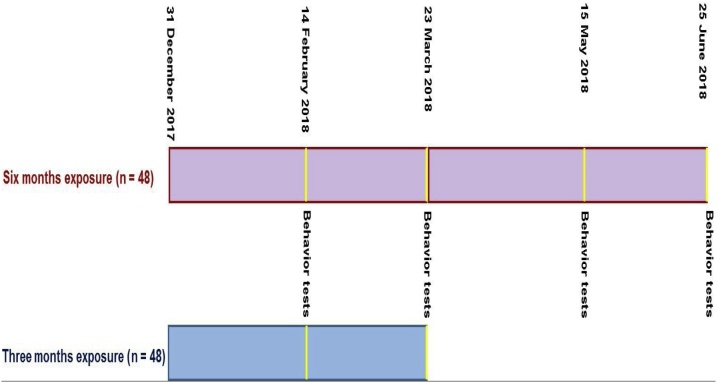
Summary of study during 3 and 6 months exposure.

A number of rats in each group was equal to 32 rats (males = 16 and females = 16). A two-period study including; 3 months (31 December, 2017–30 March, 2018) and 6 months (31 December, 2017–1 July, 2018) was selected (Fig. 2). Similar to the in-vivo model study on the neurological system effect due to air pollution [35,36], exposure time was 5 h per day (9.00 a.m.–2.00 p.m.) for 4 days per week.

### Monitoring PM_2.5_ and gaseous pollutants

The concentration of PM_2.5_ and gaseous pollutants (O_3_, NO_2_, and SO_2_) were monitored in the exposure days continuously in the ambient air of adjacent of pilot animal room. Concentration of PM_2.5_ by beta attenuation monitoring technique [37] and concentration of gaseous pollutants (O_3_, NO_2_, and SO_2_) by UV fluorescence (Horiba AP-370 series) was monitored which are certified instruments based on EU directives [38].

### Sampling of PM_2.5_

The sampling of PM_2.5_ ambient air of the pilot animal room was conducted using LVS (Echo PM) from 31 December 2017 to 1 July 2018. The sampling was performed at 20 L/min flow rate with 24 h (9.00 A.M.–9.00 A.M.). Quartz microfiber filters (47 mm diameter, Whatman International Ltd) was used for a collect of PM_2.5_. Before sampling, Quartz microfiber filters (47 mm diameter, Whatman International Ltd) was washed with distilled water and was located in an oven (105 °C, 2 h) [39]. Afterward, QM filters were subjected to relative humidity equal to 40–50% in the room temperature equal to 18–20 °C for 24 h [40]. After sampling, until analysis, to prevent evaporation and photodegradation, QM filters were stored in the −10 °C in the aluminum foil [41].

### Digestion and analysis of metals procedure

One-fourth of each QM filter was shredded into 15 mL Teflon container. Then, 2.5 mL HNO_3_ (69%) and 2.5 mL of concentrated of HClO_4_ (70%) were added in Teflon container and then at 170 °C for 4 h heated until dryness. Hence, the solution was dried at 95–100 °C on a hot plate. Afterward, 2.5 mL of HNO_3_ and 2.5 mL of distilled water were added to the samples. Samples were shaken at 180 rpm for 30 min [42]. The solutions were filtered via Whatman filter (paper No 42; 125 mm) and diluted up to 10 mL and then were stored in a plastic vial at 4 °C until analysis day. For assess of validity digestion method, blank sample (clean Whatman filter) following with other samples were digested. Based on a previously conducted method [43], metals analysis was conducted via inductively coupled plasma mass spectrometry (ICP-MS Model 7900 Agilent Technologies, Santa Clara, CA). Operating parameters of ICP-MS was presented in Table 1s.

### Digestion and analysis of PAHs procedure

One-fourth of each QM filter was shredded into 15 mL Teflon container. Then, 2.5 mL CH₃OH and 2.5 mL of CH₂Cl₂ were added in Teflon container and was sonicated in an ultrasonic bath with 20 kHz for 30 min (Elmasonic S 80H). Finally, solutions were filtered via 0.22 μm Millipore PTFE filters (Hesperia, CA, USA). The concentration of 16-PAHs was performed using gas chromatography–mass spectrometry (GC–MS) (Agilent model 5890A). Operating parameters of GC–MS presented was demonstrated in Table 2s.

### Quality assurance

#### Heavy metals

The concentration of metals in the sample was detected at 3 replications and the mean was calculated. The recovery rate of metals ranged from 83 to 98% (RSD: 2–6%). The limit of detection (LOD) and limit of quantitation (LOQ) for Pb was 5 μg/L and 1 μg/L. However, these values for other metals were 25 μg/L and 5 μg/L, respectively (Table 3s).

#### 16-PAHs

The concentration of 16-PAHs in the sample was detected at 3 replications, and the mean was calculated. The recovery rate of 16 PAHs ranged from 79 to 98% (RSD: 4–8%). PAHs were not observed in any of the analytical blanks. LOD and LOQ for 16 PAHs was <2 ng/l and <10 ng/l, respectively (Table 4s).

### Cognitive-like behavior assessment via Y maze

To investigate cognitive-like behavior, the Y maze test was conducted with at least 8 rats in groups. Y maze test was performed in the light phase of the cycle for least to stressful [44]. The custom-made Y maze has designed as a ‘Y’ shape with 3 arms (A, B and C) (10 cm wide, 60 cm long and 25 cm high) which are at 120° angle. Custom-made Y maze was located at the center of a behavior room at the low light conditions (Fig. 1, Fig. 2).

Y maze test was performed in 2 steps [45]. In step 1, rats were placed in the long arm (A-arm) with lateral closed for 20 s and in step 2, lateral A-arm is open for 8 min. Rats were excluded from the test when had lower than 12 ar m entries and/or have not new entries for 2 min during 8 min period. Healthy rat with hippocampal memory and learning have a preference for the search of new locations [46]. Hence rat with higher alternation indicates more healthy hippocampal memory and learning [46]. When rat completed entries into a new arm before returning to the visited arms successful alternation was happened [47]. The results of the test were presented as alternation (%), that was calculated by the following equation [46,47]:

(1) $$\;\text{alternation}\;=\;\frac{\text{Number}\;\text{of}\;\text{successful}\;\text{arm}\;\text{entries}\;}{\text{Total}\;\text{arm}\;\text{entries}\;-\;2}\;\times 100$$

### Cognitive-like behavior assessment via force swimming

In the forced swimming test, the duration of immobilization is equivalent to depression and its reduction as an antidepressant effect. After exposure, the rats were placed separately in a transparent cylindrical container with 30 cm diameter and 80 cm depth. The depth of water was 20 cm, and its temperature was 25 °C. Stopping the movement's hands and feet of the rat was considered as a time of immobilization. The whole test was 6 min, which was 2 min for adaptation of the rat and in the 4 min was recorded immobilization, swimming and climbing time of rats via chronometer [48].

### Statistical analysis

*Distribution of concentration of PM_2.5_*, gaseous pollutants (O_3_, NO_2_, and SO_2_), alternation and immobilization time was determined by Kolmogorov–Smirnov (KS) test. Compare concentration of *PM_2.5_*, gaseous pollutants (O_3_, NO_2_, and SO_2_) with standard limits was performed by one sample *t*-test. Difference alternation (%) and immobilization time among three groups of rats were determined by two-way analysis of variance (ANOVA) analysis with Tukey’s test. All statistical analyses and graph design were conducted using GraphPad PRISM version 5.01 software (Inc., San Diego, CA. USA). All results mentioned as the mean ± standard error of the mean (M ± SEM). If p-value <0.05 selected as statistically significant.

## Results

### The concentration of PM_2.5_, O_3_, NO_2_, and SO_2_

The concentration of PM_2.5_ in the 3 and 6 months of exposure was presented in Table 1. The mean concentration of PM_2.5_ in the 3 and 6 months exposure was 37.76 ± 11.96 and 31.61 ± 11.20 μg/m^3^, respectively. Concentration of PM_2.5_ in the 3 and 6 months exposure was higher than WHO guideline (25 μg/m^3^), significantly (p-value <0.05) [49]. The mean concentration of O_3_ in the 3 and 6 months exposure was 16.55 ± 2.30 and 23.37 ± 7.72 ppb, respectively. The concentration of O_3_ in the 3 and 6 months exposure was lower than WHO guideline (100 ppb), significantly (p-value <0.05) [49]. The mean concentration of SO_2_ in the 3 and 6 months exposure was 5.90 ± 1.30 and 5.37 ± 1.23 ppb, respectively. The concentration of SO_2_ in the 3 and 6 months exposure was lower than WHO guideline (20 ppb), significantly (p-value <0.05) [49]. The mean concentration of NO_2_ in the 3 and 6 months exposure 56.88 ± 7.82 and 53.28 ± 8.73 ppb, respectively. The concentration of NO_2_ in the 3 and 6 months exposure was lower than WHO guideline (100 ppb), significantly (p-value <0.05) [49].

**Table 1:** Concentration of PM_2.5_ and gaseous pollutants in ambient air of pilot animal room.

Contaminants	Duration of exposure	Unit	Mean	Range	WHO guideline
PM_2.5_	3 Months	μg/m^3^	37.76 ± 11.96	13.17–58.57	25
	6 Months	μg/m^3^	31.61 ± 11.20	10.01–58.57	

O_3_	3 Months	ppb	16.55 ± 2.30	12.00–23.00	100
	6 Months	ppb	23.37 ± 7.72	12.00–39.00	

SO_2_	3 Months	ppb	5.90 ± 1.30	4.00–8.00	20
	6 Months	ppb	5.37 ± 1.23	3.00–8.00	

NO_2_	3 Months	ppb	56.88 ± 7.82	43.00–77.00	100
	6 Months	ppb	53.28 ± 8.73	37.00–77.00	

In the current study, dislike gaseous pollutants, concentration of PM_2.5_ was higher than WHO guideline (Table 1) mainly due to their higher emission from sources. Emission sources of PM_2.5_ in the urban areas are including natural (dust), anthropogenic (combustion of fuel in vehicles) and marginal sources (PM of factories around the urban area) but gaseous pollutants emitted from anthropogenic sources mainly [2]. On the other hand, gaseous pollutants are rapidly converted to other compounds due to their reactivity [9,50].

### The concentration of heavy metals bound with PM_2.5_

The concentration of heavy metals bound with PM_2.5_ was presented in Table 2. The order of heavy metals bound with PM_2.5_ based on mean concentration in first 3 months exposure was ranked as Al > Ca > Cu > Cd > Na > Fe > Cr > Ni > Mn > Pb (Table 2). Also, the order of metals bound with PM_2.5_ based on mean concentration in 6 months exposure was Ca > Al > Cu > Cd > Na > Fe > Cr > Ni > Mn > Pb. The results showed that the concentration of Al was highest while compared with other metals bound PM_2.5_ after 3 months exposure while the concentration of Ca was the highest among other metals bound PM_2.5_ after 6 months exposure.

**Table 2:** Concentration of heavy metals bound PM_2.5_ in duration exposure (μg/m^3^).

Heavy metals	3 Months	6 Months
Al	68.16 ± 16.57	8.09 ± 4.15
Ca	56.50 ± 4.21	14.70 ± 12.65
Cd	1.16 ± 3.64	0.17 ± 0.10
Cr	0.57 ± 0.22	0.32 ± 0.39
Cu	1.74 ± 4.53	0.98 ± 1.28
Fe	0.20 ± 0.12	0.57 ± 0.52
Mn	0.17 ± 0.07	0.13 ± 0.06
Na	3.02 ± 3.26	0.88 ± 1.20
Ni	0.30 ± 0.10	0.18 ± 0.18
Pb	0.28 ± 0.19	0.18 ± 0.13

### The concentration of PAHs bound with PM_2.5_

The concentration of PAHs bound with PM_2.5_ was presented in Table 3. The sum concentration of 16 PAHs bound PM_2.5_ in the 3, and 6 months exposure was 45.7 ± 37.15 ng/m^3^ and 30.04 ± 25.27 ng/m^3^, respectively. The order of 16-PAHs was Phenanthrene > Naphtalene > Chrysene > Anthracene > Acenaphtylen > B(a)P > Acenaphten > Florene > Pyrene > Fluorantene > Benzo(a)ant > B(k)F ˜ Indeno(1,2,3-cd)Pyrene ˜ B(b)F ˜ Dibenzo(a,h)Anthracene ˜ Benzo g,h,iPerylene. According to findings, the concentration of Phenanthrene was higher than other PAHs bound PM_2.5_ that were inhalated by rats after both 3 and 6 months exposure.

**Table 3:** Concentration of 16-PAHs bound PM_2.5_ in two exposure periods (ng/m^3^).

16-PAHs	3 Months	6 Months
Naphtalene	7.17 ± 16.17	4.39 ± 11.4
Acenaphtylen	1.99 ± 0.8	2.57 ± 0.43
Acenaphten	2.96 ± 1.92	1.67 ± 1.34
Florene	0.96 ± 0.57	1.33 ± 0.34
Phenanthrene	18.98 ± 10.35	9.86 ± 7.26
Anthracene	5.3 ± 3.27	2.89 ± 2.3
Fluorantene	1 ± 0.57	0.86 ± 0.38
Pyrene	1.74 ± 0.78	0.87 ± 0.55
Benzo(*a*)ant	0.35 ± 0	0.35 ± 0
Chrysene	3.05 ± 1.27	3.05 ± 1.27
B(*b*)F	ND	ND
B(*k*)F	ND	ND
B(*a*)P	2.2 ± 1.45	2.2 ± 1.45
Dibenzo(*a,h*) Anthracene	ND	ND
Benzo(*g,h,i*) Perylene	ND	ND
Indeno(1,2,3-cd)Pyrene	ND	ND
**Sum**	**45.7 ± 37.15**	**30.04 ± 25.27**

### Cognitive-like behavior assessment

#### Y maze and force swimming

**Fig. 3:**
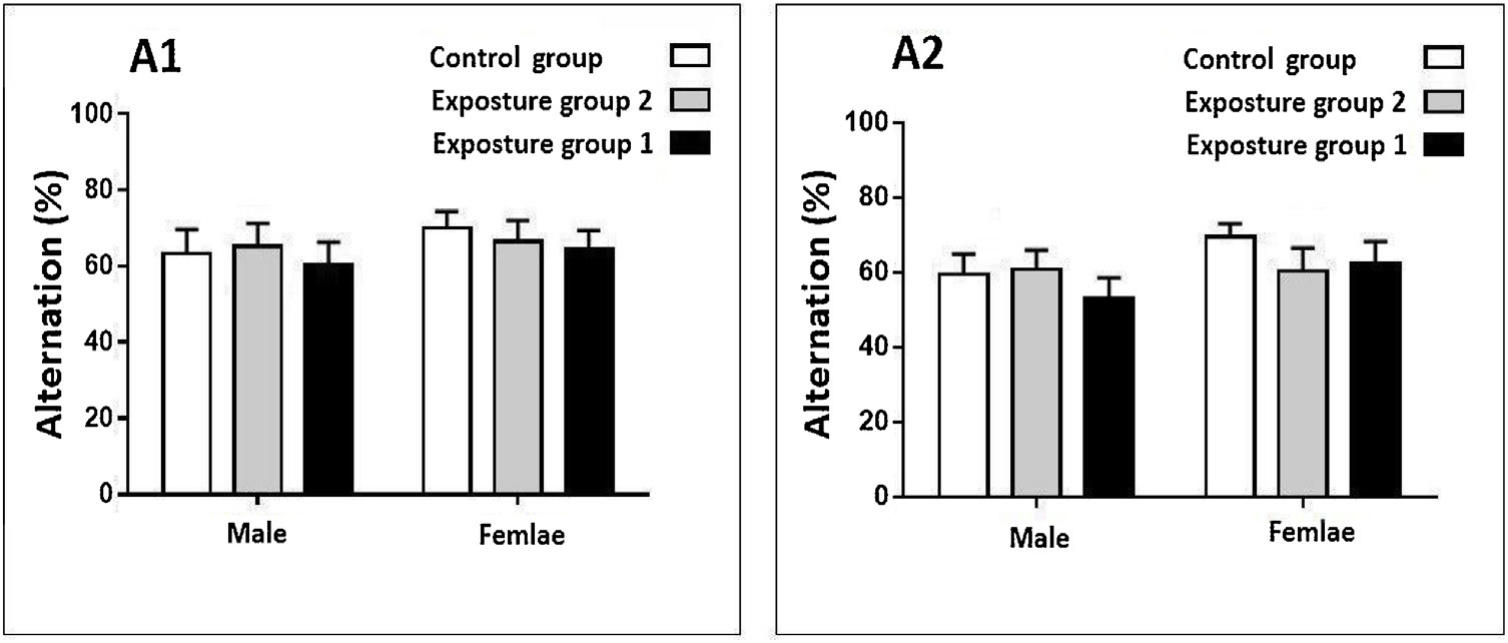
Compare changes alternation (%) in the male and female rats after 3 months exposure.

**Fig. 4:**
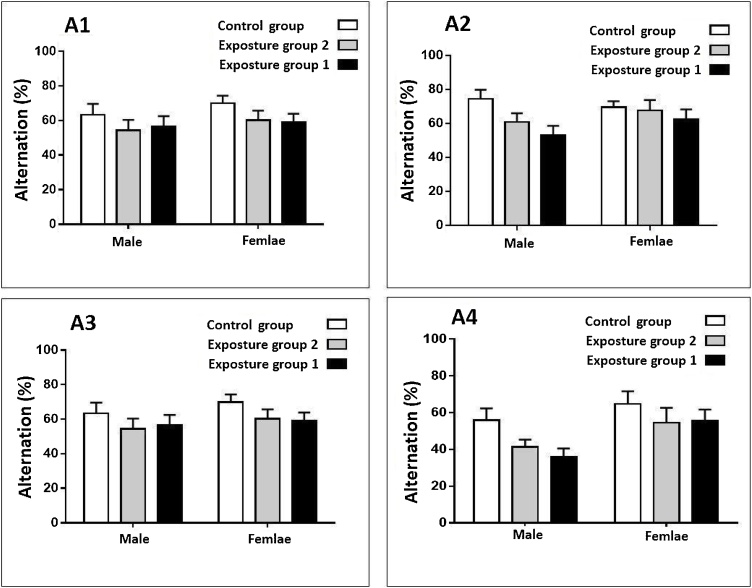
Compare changes alternation (%) in the male and female rats after 6 months exposure.

Exposure to ambient PM_2.5_ plus gaseous pollutants did not affect alternation in the male and female rats after in both 3 and 6 months periods study (p-value >0.05) (Fig. 3, Fig. 4). Exposure to gaseous pollutants alone had no significant effects on alternation in both male and female rats (p-value >0.05) (Fig. 3, Fig. 4). Also, a significant difference between alternation in male and female rats in both periods study was not observed (p-value >0.05) (Fig. 3, Fig. 4).

**Fig. 5:**
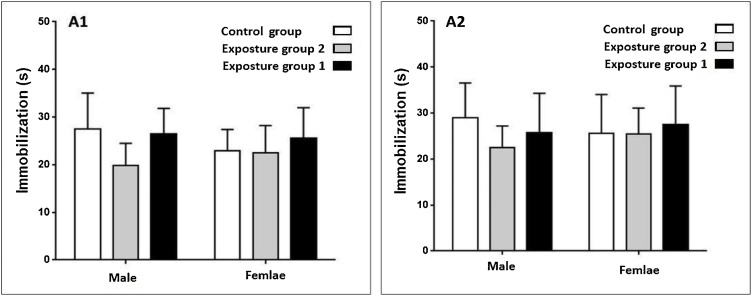
Compare changes immobilization time (s) in the male and female rats after 3 months exposure.

**Fig. 6:**
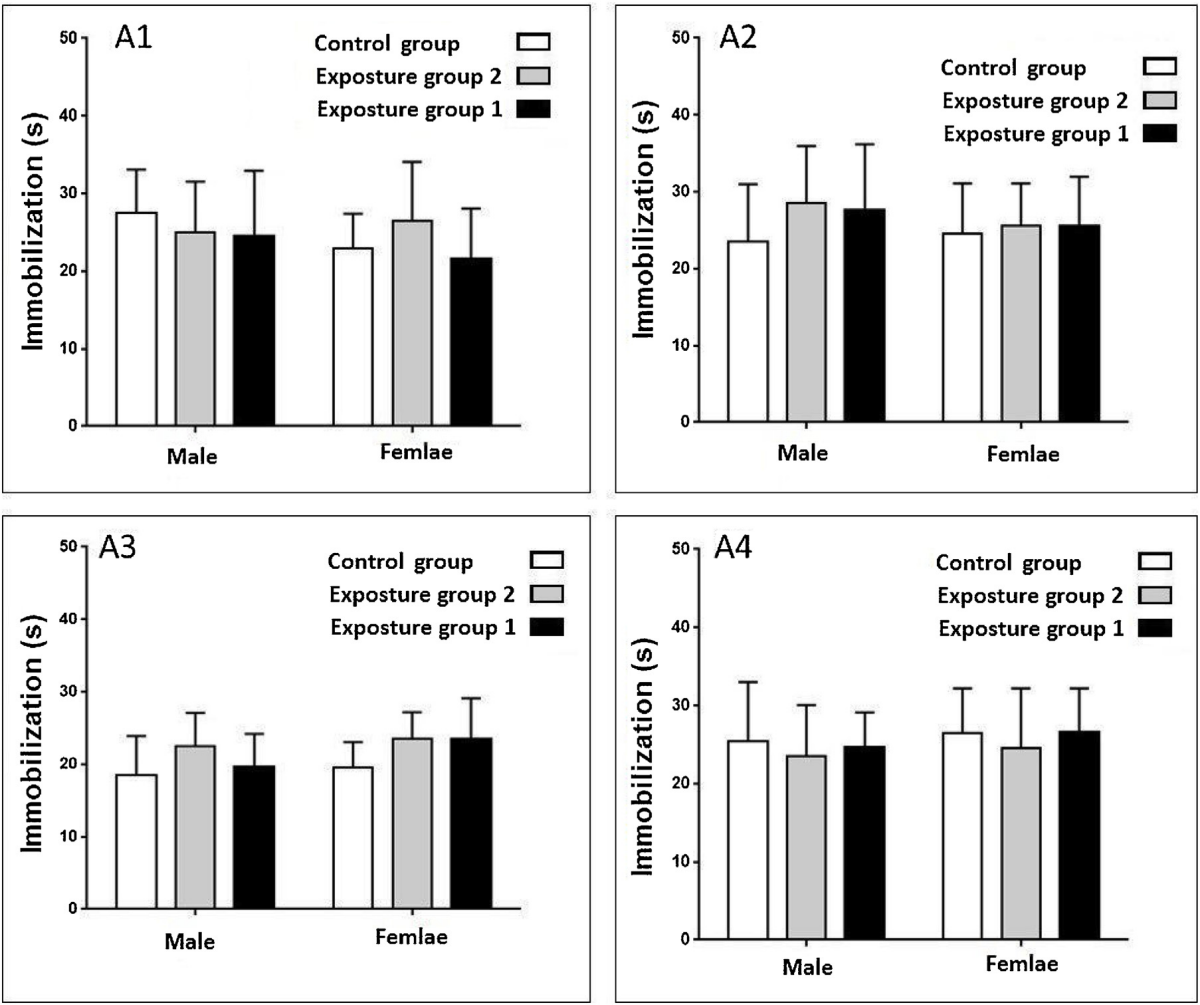
Compare changes immobilization time (s) in the male and female rats after 6 months exposure.

Exposure to ambient PM_2.5_ plus gaseous pollutants did not affect immobilization time in the male and female rats after in both 3 and 6 months periods study (p-value >0.05) (Fig. 5, Fig. 6). Exposure to gaseous pollutants alone had no significant effects on immobilization time in both male and female rats (p-value >0.05) (Fig. 5, Fig. 6). Also, a significant difference between immobilization time in male and female rats in both periods study was not observed (p-value >0.05) (Fig. 5, Fig. 6). The alternation and immobilization time in the exposed groups was not significantly higher than the control group (p-value >0.05) (Fig. 3, Fig. 4, Fig. 5, Fig. 6).

## Conclusion

In the current study, effect of exposure to PM_2.5_ and gaseous pollutants on the Alzheimer and depressive cognitive-like behaviors in the rats after 3 and 6 months of exposure by approaching a new method exposure was investigated. The concentration of PM_2.5_ in the both 3 and 6 months exposure was higher than WHO guideline, significantly (p-value <0.05) however concentration of O_3_, NO_2_, and SO_2_ was lower than the WHO guideline, significantly (p-value <0.05). Among metals bound PM_2.5_, the concentration of Al and Ca was higher than other metals and also among 16-PAHs bound PM_2.5_, the concentration of Phenanthrene was higher than other PAHs. Exposure to PM_2.5_ and gaseous pollutants separately cannot significantly increase Alzheimer and depressive cognitive-like behaviors in the rats after both 3 and 6 months exposure. Also, a significant difference between male and female in Alzheimer and depressive cognitive-like behaviors not observed.

## Conflict of interest

The authors of this article declare that they have no conflict of interests.

## References

[1] Mirzaei N., Arfaeinia H., Moradi M., Mohammadi Moghadam F., Velayati A., Sharafi K. (2015). The statistical analysis of seasonal and time variations on trend of important air pollutants (SO2, O3, NOx, CO, PM10)-in western Iran: a case study. Int. J. Pharm. Technol..

[2] Akimoto H. (2003). Global air quality and pollution. Science.

[3] Loomis D., Huang W., Chen G. (2014). The International Agency for Research on Cancer (IARC) evaluation of the carcinogenicity of outdoor air pollution: focus on China. Chin. J. Cancer.

[4] Ogino K., Zhang R., Takahashi H., Takemoto K., Kubo M., Murakami I., Wang D.-H., Fujikura Y. (2014). Allergic airway inflammation by nasal inoculation of particulate matter (PM2.5) in NC/Nga mice. PLoS One.

[5] Polichetti G., Cocco S., Spinali A., Trimarco V., Nunziata A. (2009). Effects of particulate matter (PM10, PM2.5 and PM1) on the cardiovascular system. Toxicology.

[6] Khamutian R., Najafi F., Soltanian M., Shokoohizadeh M.J., Poorhaghighat S., Dargahi A., Sharafi K., Afshari A. (2015). The association between air pollution and weather conditions with increase in the number of admissions of asthmatic patients in emergency wards: a case study in Kermanshah. Med. J. Islam. Repub. Iran.

[7] Ko F.W., Hui D.S. (2012). Air pollution and chronic obstructive pulmonary disease. Respirology.

[8] Royal College of Physicians (2016). Every Breath We Take: The Lifelong Impact of Air Pollution.

[9] Morakinyo O.M., Mokgobu M.I., Mukhola M.S., Hunter R.P. (2016). Health outcomes of exposure to biological and chemical components of inhalable and respirable particulate matter. Int. J. Environ. Res. Publ. Health.

[10] Pacitto A., Stabile L., Viana M., Scungio M., Reche C., Querol X., Alastuey A., Rivas I., Álvarez-Pedrerol M., Sunyer J. (2018). Particle-related exposure, dose and lung cancer risk of primary school children in two European countries. Sci. Total Environ..

[11] Shahsavani A., Naddafi K., Haghighifard N.J., Mesdaghinia A., Yunesian M., Nabizadeh R., Arahami M., Sowlat M., Yarahmadi M., Saki H. (2012). The evaluation of PM10, PM2.5, and PM1 concentrations during the Middle Eastern Dust (MED) events in Ahvaz, Iran, from april through september 2010. J. Arid Environ..

[12] Cohen A.J., Brauer M., Burnett R., Anderson H.R., Frostad J., Estep K., Balakrishnan K., Brunekreef B., Dandona L., Dandona R. (2017). Estimates and 25-year trends of the global burden of disease attributable to ambient air pollution: an analysis of data from the Global Burden of Diseases Study 2015. Lancet.

[13] Abtahi M., Fakhri Y., Oliveri Conti G., Ferrante M., Taghavi M., Tavakoli J., Heshmati A., Keramati H., Moradi B., Amanidaz N. (2018). The concentration of BTEX in the air of Tehran: a systematic review-meta analysis and risk assessment. Int. J. Environ. Res. Publ. Health.

[14] Rui W., Guan L., Zhang F., Zhang W., Ding W. (2016). PM2.5‐induced oxidative stress increases adhesion molecules expression in human endothelial cells through the ERK/AKT/NF‐κB‐dependent pathway. J. Appl. Toxicol..

[15] Rezaei M., Salimi A., Taghidust M., Naserzadeh P., Goudarzi G., Seydi E., Pourahmad J. (2014). A comparison of toxicity mechanisms of dust storm particles collected in the southwest of Iran on lung and skin using isolated mitochondria. Toxicol. Environ. Chem..

[16] Yegambaram M., Manivannan B., Beach T.G., Halden R.U. (2015). Role of environmental contaminants in the etiology of Alzheimer’s disease: a review. Curr. Alzheimer Res..

[17] Lemos A.T., de Lemos C.T., Flores A.N., Pantoja E.O., Rocha J.A.V., Vargas V.M.F. (2016). Genotoxicity biomarkers for airborne particulate matter (PM2.5) in an area under petrochemical influence. Chemosphere.

[18] Tyler C.R., Noor S., Young T.L., Rivero V., Sanchez B., Lucas S., Caldwell K.K., Milligan E.D., Campen M.J. (2018). Aging exacerbates neuroinflammatory outcomes induced by acute ozone exposure. Toxicol. Sci..

[19] McKhann G.M., Knopman D.S., Chertkow H., Hyman B.T., Jack C.R., Kawas C.H., Klunk W.E., Koroshetz W.J., Manly J.J., Mayeux R. (2011). The diagnosis of dementia due to Alzheimer’s disease: recommendations from the National Institute on Aging-Alzheimer’s Association workgroups on diagnostic guidelines for Alzheimer’s disease. Alzheimer’s Dement..

[20] Alzheimer’s Association (2018). 2018 Alzheimer’s disease facts and figures. Alzheimer’s Dement..

[21] Tang-Wai D.F., Josephs K.A., Petersen R.C. (2005). Alzheimer’s disease–overview, neurodegenerative diseases: neurobiology. Pathog. Ther..

[22] Viña J., Lloret A. (2010). Why women have more Alzheimer’s disease than men: gender and mitochondrial toxicity of amyloid-β peptide. J. Alzheimers Dis..

[23] Zigman W.B., Lott I.T. (2007). Alzheimer’s disease in Down syndrome: neurobiology and risk. Ment. Retard. Dev. Disabil. Res. Rev..

[24] Newman A.B., Fitzpatrick A.L., Lopez O., Jackson S., Lyketsos C., Jagust W., Ives D., DeKosky S.T., Kuller L.H. (2005). Dementia and Alzheimer’s disease incidence in relationship to cardiovascular disease in the Cardiovascular Health Study cohort. J. Am. Geriatr. Soc..

[25] Hsu H.-W., Bondy S.C., Kitazawa M. (2018). Environmental and dietary exposure to copper and its cellular mechanisms linking to Alzheimer’s disease. Toxicol. Sci..

[26] Weuve J., Puett R.C., Schwartz J., Yanosky J.D., Laden F., Grodstein F. (2012). Exposure to particulate air pollution and cognitive decline in older women. Arch. Intern. Med..

[27] Lim Y.-H., Kim H., Kim J.H., Bae S., Park H.Y., Hong Y.-C. (2012). Air pollution and symptoms of depression in elderly adults. Environ. Health Perspect..

[28] Grippo A.J. (2009). Mechanisms underlying altered mood and cardiovascular dysfunction: the value of neurobiological and behavioral research with animal models. Neurosci. Biobehav. Rev..

[29] Wang Q., Yang Z. (2018). Does chronic disease influence susceptibility to the effects of air pollution on depressive symptoms in China?. Int. J. Ment. Health Syst..

[30] Qian Z., Chapman R.S., Tian Q., Chen Y., Lioy P.J., Zhang J. (2000). Effects of air pollution on children’s respiratory health in three Chinese cities. Arch. Environ. Health Int. J..

[31] Bhatt D.P., Puig K.L., Gorr M.W., Wold L.E., Combs C.K. (2015). A pilot study to assess effects of long-term inhalation of airborne particulate matter on early Alzheimer-like changes in the mouse brain. PLoS One.

[32] Alessandria L., Schilirò T., Degan R., Traversi D., Gilli G. (2014). Cytotoxic response in human lung epithelial cells and ion characteristics of urban-air particles from Torino, a northern Italian city. Environ. Sci. Pollut. Res..

[33] AWR (2013). Animal Welfare Act.

[34] TCR (2014). Echo TCR Tecora SamplerLow Volume Sampler for PM10 and PM2.5. http://www.tecora.com/en/echo-pm/1114-echo-pm.html.

[35] Jang S., Kim E.W., Zhang Y., Lee J., Cho S.Y., Ha J., Kim H., Kim E. (2018). Particulate matter increases beta-amyloid and activated glial cells in hippocampal tissues of transgenic Alzheimer’s mouse: involvement of PARP-1. Biochem. Biophys. Res. Commun..

[36] Hernández-Zimbrón L.F., Rivas-Arancibia S. (2016). Syntaxin 5 overexpression and β-amyloid 1–42 accumulation in endoplasmic reticulum of hippocampal cells in rat brain induced by ozone exposure. BioMed Res. Int..

[37] Triantafyllou E., Diapouli E., Tsilibari E.M., Adamopoulos A.D., Biskos G., Eleftheriadis K. (2016). Assessment of factors influencing PM mass concentration measured by gravimetric & beta attenuation techniques at a suburban site. Atmos. Environ..

[38] Kattner L., Mathieu-Üffing B., Burrows J., Richter A., Schmolke S., Seyler A., Wittrock F. (2015). Monitoring compliance with sulfur content regulations of shipping fuel by in situ measurements of ship emissions. Atmos. Chem. Phys..

[39] Sowlat M.H., Naddafi K., Yunesian M., Jackson P.L., Shahsavani A. (2012). Source apportionment of total suspended particulates in an arid area in southwestern Iran using positive matrix factorization. Bull. Environ. Contam. Toxicol..

[40] EPA (1999). Compendium of Methods for the Determination of Inorganic Compounds in Ambient Air; EPA/625/R-96/010a. Selection, Preparation and Extraction of Filter Material.

[41] EPA (2017). Method 201a—Determination of Pm 10 and Pm 2.5 Emissions from Stationary Sources (constants Sampling Rate Procedure).

[42] Sowlat M.H., Naddafi K., Yunesian M., Jackson P.L., Lotfi S., Shahsavani A. (2013). PM10 source apportionment in Ahvaz, Iran, using positive matrix factorization. Clean–Soil Air Water.

[43] Pérez N., Pey J., Querol X., Alastuey A., López J., Viana M. (2008). Partitioning of major and trace components in PM10–PM2.5–PM1 at an urban site in Southern Europe. Atmos. Environ..

[44] Camara M.L., Corrigan F., Jaehne E.J., Jawahar M.C., Anscomb H., Koerner H., Baune B.T. (2013). TNF-α and its receptors modulate complex behaviours and neurotrophins in transgenic mice. Psychoneuroendocrinology.

[45] Choy K.H.C., de Visser Y., Nichols N.R., van den Buuse M. (2008). Combined neonatal stress and young‐adult glucocorticoid stimulation in rats reduce BDNF expression in hippocampus: effects on learning and memory. Hippocampus.

[46] Dulawa S.C., Grandy D.K., Low M.J., Paulus M.P., Geyer M.A. (1999). Dopamine D4 receptor-knock-out mice exhibit reduced exploration of novel stimuli. J. Neurosci..

[47] Yadav R., Hillman B.G., Gupta S.C., Suryavanshi P., Bhatt J.M., Pavuluri R., Stairs D.J., Dravid S.M. (2013). Deletion of glutamate delta-1 receptor in mouse leads to enhanced working memory and deficit in fear conditioning. PLoS One.

[48] Borsini F., Meli A. (1988). Is the forced swimming test a suitable model for revealing antidepressant activity?. Psychopharmacology (Berl.).

[49] WHO (2009). Update on WHO Air Quality Guidelines. http://www.euro.who.int/en/health-topics/environment-and-health/air-quality/publications/journal-articles/update-on-who-air-quality-guidelines.

[50] Schnelle K.B., Dunn R.F., Ternes M.E. (2015). Air Pollution Control Technology Handbook.

