# Supplementary material for: Alzheimer and depressive cognitive-like behaviors in male and female rats: A new method for exposure to ambient air pollution
Source: MethodsX. 2019 Mar 28;6:690–703. doi: 10.1016/j.mex.2019.03.018 (PMC6454125; doi:10.1016/j.mex.2019.03.018)
Supplement: Supplementary file 1 [file mmc1.docx]

**Table 1s.** Operating parameters of ICP-MS for the analysis of metal

| **Parameter** | Value | Unit |
| --- | --- | --- |
| Plasma argon flow | 12 | L/min |
| Sample flow rate | 1 | mL/min |
| Nebulizer argon flow | 0.7 | L/min |
| Nebulizer argon flow | 0.7 | L/min |
| Auxiliary argon flow | 0.3 | L/min |
| Sample uptake time | 240 | s |
| Measurement replicate | 3 | - |
| Type of detector Solid state | CCD | - |
| Plasma, auxiliary and nebulizer gas | Argon | - |

**Table 2s.** Operating parameters of GC/MS or the analysis of PAHs

| **Parameter** | **Value** | **Unit** |
| --- | --- | --- |
| Injection volume | 1 | µl |
| Temperature in splitless mode | 290 | °C |
| Carrier gas | Helium | - |
| Flow rate | 1 | mL/min |
| Held time in 100 °C | 1 | min |
| Increase temperature rate to 285 °C | 4 | °C/min |
| Final temperature | 285 | °C/min |
| Initial temperature | 60 | °C |
| Increase temperature rate to 100 °C | 10 | °C/min |

Table 3s. LOD (μg/L), LOQ (μg/L), recoveries (%) and relative standard deviations (%) of heavy metals analyzed by ICP-MS at 6 spiking levels (n = 3)

| Metal | LOD | LOQ | Recoveries (n = 3) | Mean of RSD (n = 3) | Regression coefficient (R^2^) |
| --- | --- | --- | --- | --- | --- |
| Pb | 1 | 5 | 95 (93-114) | 2.22 | 0.9985 |
| Cd | 5 | 25 | 83 (80- 111) | 5.45 | 0.9965 |
| Cr | 5 | 25 | 95 (92-104) | 5.65 | 0.9989 |
| Fe | 5 | 25 | 98 (95-115) | 4.45 | 0.9973 |
| Al | 5 | 25 | 95 (93-104) | 4.65 | 0.9986 |
| Cu | 5 | 25 | 96 (92-100) | 5.56 | 0.9903 |
| Mn | 5 | 25 | 96 (91-102) | 6.98 | 0.9976 |
| Ni | 5 | 25 | 98 (95-109) | 3.54 | 0.9997 |
| Ca | 5 | 25 | 94 (91-100) | 4.32 | 0.9974 |
| Na | 5 | 25 | 97 (98-107) | 5.65 | 0.9974 |

Table 4s. LOD (ng/L), LOQ (ng/L), recoveries (%) and relative standard deviations (%), enrichment factor and regression coefficient of 16 PAHs compounds analyzed by GC/MS

| **PAHs compounds** | **LOD (ng/L)** | **LOQ (ng/L)** | **Recoveries**  **(n = 3)** | **Mean of RSDr (n = 3)** | **Enrichment Factor** | **Regression coefficient (R^2^)** |
| --- | --- | --- | --- | --- | --- | --- |
| Naphtalene | 2 | 10 | 87 (85-98) | 5 | 79 | 0.9989 |
| Acenaphtylen | 2 | 10 | 89 (83-97) | 5 | 77 | 0.9976 |
| Acenaphten | 2 | 10 | 79 (77-99) | 4 | 75 | 0.9908 |
| Florene | 2 | 10 | 93 (90-105) | 4 | 73 | 0.9956 |
| Phenanthrene | 2 | 10 | 95 (91-109) | 5 | 73 | 0.9988 |
| Anthracene | 2 | 10 | 95 (90-114) | 6 | 76 | 0.9973 |
| Fluorantene | 2 | 10 | 95 (93-108) | 8 | 74 | 0.9968 |
| Pyrene | 2 | 10 | 95 (91-111) | 7 | 69 | 0.9978 |
| Benzo(a)ant | 2 | 10 | 96 (93-116) | 5 | 75 | 0.9909 |
| Chrysene | 2 | 10 | 96 (94-112) | 4 | 78 | 0.9984 |
| B(b)F | 2 | 10 | 98 (95-117) | 5 | 79 | 0.9947 |
| B(k)F | 2 | 10 | 98 (94-115) | 4 | 80 | 0.9968 |
| B(a)P | 2 | 10 | 92 (88-103) | 6 | 74 | 0.9971 |
| Dibenzo(a,h)Anthracene | 2 | 10 | 95 (92-106) | 5 | 75 | 0.9969 |
| Benzo(g,h,i)Perylene | 2 | 10 | 95 (90-112) | 4 | 76 | 0.9974 |
| Indeno(1,2,3-cd) Pyrene | 2 | 10 | 95 (91-109) | 5 | 69 | 0.9967 |
